# Editorial

**Published:** 1987-08

**Authors:** Huw Griffith


### Editorial

It is an astonishing fact that in the BMJ of January 17th, 1987, the BMA Council was reported as endorsing The Third BMA Statement on AIDS, agreeing that 'AIDS and HIV infection should not be made notifiable diseases'. All doctors to whom I have reported this have reacted in the same way—with incredulity. I checked the facts. There is no mistake.

What has happened? Most doctors believe that it is tantamount to sending the armed forces and intelligence services home at the outbreak of war. Faced with the greatest disease threat to the human race worldwide we are throwing away the scientific and public health weapons we have to control infectious disease. The initiative of the distinguished epidemiologist, Sir Richard Doll, in calling for population studies of HIV infection has also been stifled.

Why has this come about? Probably because we have too rapidly jumped to the conclusion that HIV is simply another and rather nastier venereal disease. The pattern of disease spread in Africa makes this unlikely. Certainly anal penetration in male homosexuality is the most easily documented mode of spread, but Dr. John Seal in this issue makes it clear that this is likely to be far from the whole story. Female prostitutes on the Ivory Coast are infected according to social class, with the lowest class having the highest infection rate. Venereal diseases are classically dealt with in our society without making them notifiable, not a particularly successful manoeuvre when this category of disease is seen to be steadily increasing. No venereal disease has ever approached AIDS/HIV in its threat.

Caring for those who are to die of HIV is humane and correct. In our society to do otherwise is simply not acceptable, even though most understandably find drug addiction and homosexuality repugnant. Hand-wringing concern, however, is simply not enough. History, nor the people of this land, will not forgive those Nero's who fiddle while Rome burns. There is little doubt that the homosexual lobby (e.g. the Terence Higgins' Trust) is concerned to conceal from our population the true state of affairs. In this it is both mistaken and anti-social as the major sufferers, if the disease is not controlled, will be the homosexuals. In this not only has it become a political force acting against the best interests of its own deviant members, but certainly against the best interests of the normal community at large. The question of its continuation of charitable status certainly arises.

Government policy so far has consisted in alarming both politicians and the public. There is as yet no evidence that this will be anything like enough nor that it will reach or touch the main reservoirs (drug addicts and homosexuals) from which the disease is now spreading. HIV infection is doubling every eight months (DHSS figures). The medical profession must make it clear that both BMA and DHSS must behave more responsibly. As a first step to doing anything effective this dreadful disease must be made notifiable. To do less will be to sacrifice many lives to the distorted views of a small and abnormal minority.

*Huw Griffith*

